# **Learning by Doing: Accelerate Towards the NCD Target in SDG Through Primary Healthcare** Comment on "Universal Health Coverage for Non-communicable Diseases and Health Equity: Lessons From Australian Primary Healthcare"

**DOI:** 10.34172/ijhpm.2021.96

**Published:** 2021-08-21

**Authors:** Cherian Varghese, Baridalyne Nongkynrih, Bente Mikkelsen

**Affiliations:** ^1^Department of Noncommunicable Diseases, World Health Organization, Geneva, Switzerland.; ^2^Centre for Community Medicine, All India Institute of Medical Sciences, New Delhi, India.

**Keywords:** Non-communicable Diseases, Primary Healthcare, Sustainable Development Goals, Local Solutions

## Abstract

Health systems built on the foundation of primary healthcare (PHC) are essential to achieve universal health coverage (UHC). To adequately respond to the needs of people with non-communicable diseases (NCDs) and enable optimal management in primary care settings, changes are needed at many levels. PHC levers recommended in the UHC framework as the cornerstone of achieving Sustainable Development Goal (SDG) goals by strengthening the primary care system include strategic and operational levers. Experience from hypertension control programs across 18 countries has shown that rapid scale-up can be achieved through systematic improvement of the PHC system brought about by political commitment, financial support, and high-quality people-centred primary care. As countries are gripped with the pandemic the importance of an appropriate and resilient health system fit for the country is emerging as a priority for building preparedness. While there are general principles, each country must learn by doing and scale up models relevant to the national context.

 Fisher et al investigated the implementation of Australian primary healthcare (PHC) policy over 10 years (between 2008 and 2018). Their analysis brings out lessons for universal health coverage (UHC) policies and the role of PHC, to address non-communicable diseases (NCDs) and promote health equity.^1^ They note that the Australian PHC system only partly supports equity and funds mainly episodic primary medical care. The authors note that the mix of public and private insurance for UHC in Australia is unfavourable to equity and suggest that publicly funded and managed health systems are more likely to achieve equitable UHC.

 Fisher and colleagues highlight the importance of actors in shaping policies for PHC. They have used a qualitative case study methodology and brought out factors determining policy and the main actors. There are learnings that we can use in these domains. Given the high coverage of UHC in Australia, the authors could have reflected on the cost of health and how the high coverage was achieved. Outcomes such as control of hypertension are tracers that can reflect the coverage and equity of PHC. A mixed method with some empirical data can provide an insight into the inputs that is needed in terms of resources. It is important to recognize the need for equitable coverage and hence the policy making must be broad based and inclusive.

 The Right to Health was first articulated in the 1946 constitution of the World Health Organization (WHO) with the definition of health in the preamble which also mentioned ‘the enjoyment of the highest attainable standards of health is one of the fundamental rights of every human being without distinction of race, religion, political belief, economic or social condition.’ Article 25 of the 1948 Universal Declaration of Human Rights also mentioned health as part of the right to an adequate standard of living.

 Seventy-five years after the proclamation, the world is struggling to provide the healthcare that people need without financial hardships. This is not just an economic argument, but a collective will of the society that has to transform the health systems and services for equitable access.

 PHC is valid for countries at all levels of income and other resources. The stress test of coronavirus disease 2019 (COVID-19) pandemic dented the comfort levels of high performing hospital systems in high income countries making everyone to introspect. Health and wellness are not commercial activities and will need to take a rights-based approach even in settings where the outcome is measured in economic terms.

 COVID-19, in a way, indicated the approach to UHC: provide testing, triaging, tracing and treatment for everyone who needs it. Primarily driven by the need to control the spread of the pandemic and to reduce the mortality numbers, but also demonstrating that unless everyone who needs the services are provided, no one can remain safe. NCDs emerged as the cornerstone of the COVID-19 pandemic. There is a deadly interplay between NCDs and COVID-19 and the service disruptions leave people undiagnosed, untreated, uncontrolled and unprotected against risk factors.^2^ People living with NCDs should be given priority, be it for prevention including vaccination and ensure that they have uninterrupted treatment.

 The income divides across the world also led to a “disease divide” whereby low-income settings require communicable diseases’ control for which high income countries will provide funding. This may be because communicable diseases anywhere are a potential threat to everyone. The other side of the story is that NCDs are mostly seen in high-income settings and insurance takes care of the expensive interventions for those who can afford them. It is this paradigm that has to change and UHC is the platform to make this change palpable to the society.

 Sustainable Development Goal (SDG) targets on UHC (3.8.1 and 3.8.2)^3^ provide a measure and plotting the progress over the years and across the domains has helped to reveal the situation. The UHC service coverage index has been developed with NCD as one of the domains.^4^ UHC monitoring report of 2019 shows the NCD domain remaining as a plateau against a substantial increase in coverage in infectious disease and maternal and child health domains.^5^ It is essential that communicable diseases and maternal and child health issues are addressed adequately; however, we do not have the luxury of completing one area and then moving into other areas. This is a realization that needs to come at all levels of the society and in the global health narrative.

 Peiris et al have published an extensive review of best practices to strengthen PHC.^6^ They have identified governance and leadership and workforce as critical inputs. Community participation, care packages and decentralized management were identified as determinants of success. Equal emphasis must be given to physical infrastructure such as electricity, medical supplies and transportation.

 The WHO PHC framework proposes strategic levers and operational levers.^7^ The four core strategic levers comprise political commitment and leadership, governance and policy frameworks, funding and allocation of resources, and the engagement of communities and other stakeholders. Operational levers include models of care that promote high-quality, people-centred primary care, engagement with private sector providers, digital technologies for health and monitoring and evaluation.

 To adequately respond to the needs of people with NCDs and enable optimal management in primary care settings, changes are needed at many levels.^8^ Multidisciplinary teams with diverse competencies; availability of essential diagnostic tools and medications as per protocols, prescription rights for non-physician care providers, use of telemedicine and digital health for service delivery, better mechanisms for referral and improved health information systems are needed to improve coverage and access to care. Community-based models of care including self-care can augment this model and NCD care should be integrated into existing programmes for tuberculosis, HIV, and maternal health services where appropriate.

 The experience of protocol-based hypertension management for 3 million people in 18 countries that have adopted the WHO HEARTS technical package has demonstrated the feasibility of a rapid scale-up of effective hypertension control programs. These programs have set a new standard for scalable public health hypertension control through the PHC system. Five components have been shown to be necessary for a successful hypertension control programme: drug- and dose-specific treatment protocols; access to quality-assured medications and blood pressure monitors; team-based care; patient-centred care delivered in the community, and information systems to enable quality improvement.^9^Investments in health systems development and the UHC scheme have greatly contributed to increasing access to care, reducing financial risk and improving equity in Thailand.^10^

 Primary care is more than a first point of care – it is the core component of a health system. It should be accessible to all patients and can undertake the management of early stages of NCDs by providing continuity of care. Primary care becomes an effective way to manage NCDs when it moves from delivering an episode of care to providing an integrated approach that includes prevention, diagnosis, treatment, and palliative care for all conditions and over time.^11^

 As countries are gripped with the pandemic the importance of an appropriate and resilient health system fit for the country is emerging as a priority. This is the right time to learn from what worked and to be bold in moving towards equitable PHC which includes NCDs as a critical component. While there are general principles, each country has to learn by doing and scale up models relevant to the national context.

## Ethical issues

 Not applicable.

## Competing interests

 Authors declare that they have no competing interests.

## Authors’ contributions

 Conception and design: CV. Acquisition of data: BN. Drafting of the manuscript: CV, BN, BM. Critical revision of the manuscript: CV, BN, BM.

## Disclaimer

 The views expressed in this commentary are solely the responsibility of the authors and they do not necessarily reflect the views, decisions, or policies of the institutions with which they are affiliated.

## Authors’ affiliations

^1^Department of Noncommunicable Diseases, World Health Organization, Geneva, Switzerland. ^2^Centre for Community Medicine, All India Institute of Medical Sciences, New Delhi, India.
